# Aurora A controls CD8^+^ T cell cytotoxic activity and antiviral response

**DOI:** 10.1038/s41598-019-38647-y

**Published:** 2019-02-18

**Authors:** Eugenio Bustos-Morán, Noelia Blas-Rus, Ana Alcaraz-Serna, Salvador Iborra, José González-Martínez, Marcos Malumbres, Francisco Sánchez-Madrid

**Affiliations:** 1 Servicio de Inmunología, Hospital Universitario de la Princesa, Universidad Autónoma de Madrid, Instituto Investigación Sanitaria Princesa (IIS-IP), Madrid, Spain; 2 Centro Nacional Investigaciones Cardiovasculares (CNIC), Madrid, Spain; 3 Department of Immunology, Ophthalmology and ENT. Complutense University School of Medicine and 12 de Octubre Health Research Institute (imas12), Madrid, Spain; 4 Cell Division and Cancer group, Centro Nacional de Investigaciones Oncológicas (CNIO), Madrid, Spain; 5 CIBERCV, Madrid, Spain

## Abstract

Aurora A is a serine/threonine kinase whose role in cell cycle progression and tumour generation has been widely studied. Recent work has revealed an unexpected function for Aurora A during CD4^+^ T cell activation and, also, in graft versus host disease development. However, it remains unknown whether Aurora A is involved in CD8^+^ T cell effector function and in cytotoxic T lymphocyte-mediated antiviral response. Here, we show that Aurora A chemical inhibition leads to an impairment of both the peptide-specific cytotoxicity and the degranulation activity of CD8^+^ T cells. This finding was similarly proven for both mice and human CD8^+^ CTL activity. As a result of Aurora A blockade, we detected a reduction in the expression induced by T cell activation of genes classically related to the effector function of cytotoxic T lymphocytes such as *granzyme B* or *perforin1*. Finally, we have found that Aurora A is necessary for CD8^+^ T cell-mediated antiviral response, in an *in vivo* model of vaccinia virus infection. Thus, we can conclude that Aurora A activity is, indeed, needed for the proper effector function of cytotoxic T lymphocytes and for their activity against viral threats.

## Introduction

Aurora A, a serine/threonine kinase involved in cell cycle progression, has mainly been studied in the context of cell division and tumorigenesis^1–3^. Aurora A belongs to a family of kinases that includes two other members, Aurora B and Aurora C. Aurora A and B share a 70% similarity but their functions and localization differ. While Aurora A decorates the centrosomes and spindle microtubules during cell division, participating in the maturation of the centrosomes, Aurora B binds to the kinetochores acting on chromosome segregation^4,5^.

Recently, new roles associated with the immune response have been reported for Aurora A. This protein plays an essential role in CD4^+^ T cells activation^6^. During this process, Aurora A acts through two different but related cellular and molecular mechanisms. Aurora A promotes the phosphorylation, and thus the activation of the Lck kinase, while, in parallel, it enhances proper Microtubule (MT) polymerization from the centrosome, allowing the movement of CD3ζ-bearing intracellular vesicles towards the Immune Synapse (IS) platform^6^.

Additionally, Aurora A has been considered as a new target for preventing graft versus host disease (GVHD)^7,8^. Aurora A expression is augmented during GVHD development and it correlates with the outcome of the disease^8^. Moreover, its blockade leads to an increase in the generation of inducible regulatory T cells (iTregs), essential for GVHD clinical improvement^7^.

Although TCR signalling pathways are shared between CD4^+^ and CD8^+^ T cells, the effector function of both subsets differs. CD4^+^ effector T cells are mainly involved in the stimulation and coordination of other immune cells, while CD8^+^ effector T cells (CTLs) mostly carry out a cytotoxic function^9^. TCR activation in CD8^+^ T cells leads to the polarized release of lytic granules containing molecules, such as perforin and granzyme B, involved in killing infected target cells, which is essential for the defence of the organism against intracellular pathogens, like viruses^10,11^.

We have assessed whether Aurora A plays a role in CD8^+^ T lymphocytes cytotoxic activity and their ability to respond against viruses. In this study, we show that Aurora A inhibition reduces the cytotoxic and degranulation capacity of human and mouse CD8^+^ T cells. Furthermore, Aurora A pharmacological blockade impairs the upregulated expression of cytotoxicity related genes and TCR downstream signalling. This reduction in all the cytotoxic features decreases the ability of CD8^+^ T cells to respond against vaccinia infection in an *in vivo* mouse model.

## Results and Discussion

### Aurora A regulates CD8^+^ T cell-mediated cytotoxicity

**Figure 1:**
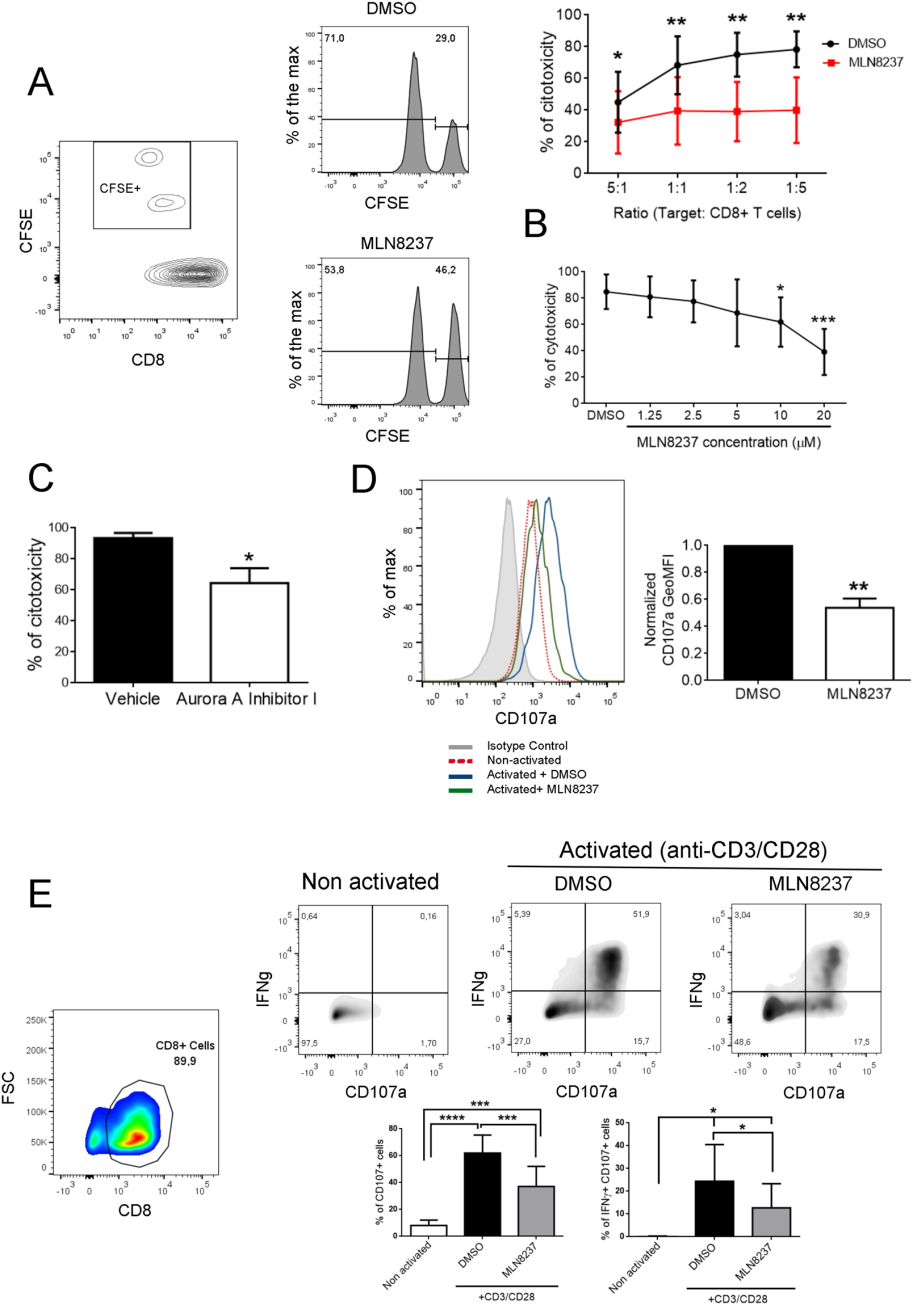
Aurora A blockade impairs cytotoxicity and degranulation capacity. (**A**) Density plot and histograms showing the gating strategy for a cytotoxicity assay from, vehicle or MLN8237-treated (10 µM), mouse OTI-CD8^+^ T cells cocultured for 6 h with unpulsed (CFSE-*low*) or OVAp-pulsed (CFSE-*high*) EL4 target cells. Cocultures were performed at different ratios and the percentage of specific lysis was quantified as in the *upper right* graph (n = 7 mice samples, paired t-test). **(B)** Quantification of the percentage of specific lysis, as described in Materials and Methods, in a cytotoxic assay with different doses of MLN8237 treatment (n = 5 mice samples, Friedman test against vehicle-treated). **(C)** Quantification of the percentage of specific lysis, as described in Materials and Methods, in a cytotoxic assay comparing vehicle or Aurora A Inhibitor I-treated (1 µM) mice (n = 3 mice samples, paired t-test). **(D)** Histogram showing CD107a fluorescence intensity of, vehicle or MLN8237-treated (10 µM) OTI-CD8^+^ cells after 6 h of coculture with unpulsed (non-activated) or pulsed (activated) EL4 in the presence of monensin. CD107a geometric mean was quantified and normalised to vehicle-treated as indicated in the graph (*right panel*; n = 8 Wilcoxon Signed Rank test). **(E)** Dot plots and density plots showing the gating strategy to analyse IFNγ *vs* CD107a expression in human CD8^+^ lymphoblasts pretreated with vehicle or MLN8237 (10 µM) and activated for 6 h with anti-CD3/CD28 coated plates in the presence of monensin. Quantification of the % of CD107a^+^ cells as well as CD107a^+^/IFNγ^+^ is shown below (n = 6 human samples, ANOVA t-test and Wilcoxon test, respectively). P-value: < 0.05*; < 0.01**; < 0.0001****. Mean ± s.d.

In order to assess the role of Aurora A in CD8^+^ T cell-mediated cytotoxic response, OTI mouse T lymphoblasts were cocultured for 6 h with target cells (EL4 cell line) in the presence of Aurora A specific inhibitor (MLN8237) or vehicle (DMSO). Target cells were previously pulsed with the H-2 Kb-restricted Ovalbumin peptide (257–264; OVAp), or left unpulsed; stained with CFSE (1 and 0.1 µM, respectively) and mixed in a 1:1 ratio. A significant decrease in the percentage of cytotoxicity was detected as a result of Aurora A blockade (Fig. 1A). This impairment in the cytotoxic activity was similarly detected by using different ratios of T cells *vs* target cells (Fig. 1A). Furthermore, when different dosages of Aurora A inhibitor were applied, only doses up to 10 μM or higher were able to significantly reduce cytotoxicity (ratio 1:5) (Fig. 1B). Likewise, the application of a different Aurora A inhibitor (Aurora A inhibitor I), also caused a significant decrease on the cytotoxic capacity (ratio 1:5) of CD8^+^ T cells (Fig. 1C).

As a complementary approach, the effect of Aurora A inhibition on T cell degranulation activity was measured. The expression of CD107a in CD8^+^ mouse (Fig. 1D) or human T lymphoblasts (Fig. 1E) was assessed by flow cytometry staining, in the presence of Aurora A inhibitor (MLN8237) or vehicle. Mouse CD8^+^ T cells were cocultured for 6 h with OVAp-pulsed EL4 mice cells, while human T lymphoblasts were incubated for 6 h with anti-human CD3/CD28 antibodies. Flow cytometry analysis showed a significant reduction in the degranulation rate of MLN8237-treated cells in mice (Fig. 1D). Likewise, treatment of human CD8^+^ T cells activated with anti-CD3/CD28 antibodies with MLN8237 resulted in a decrease in both the percentage of CD107^+^ T cells and the frequency of CD107^+^ IFNγ^+^ cells (Fig. 1E). As a control, and taking into account the sequence similarity between Aurora A and Aurora B, both the cytotoxicity (Supp. Fig. 1A) and the degranulation capacity (Supp. Fig. 1B) of CD8^+^ mouse T cells was, in a similar manner, studied in the presence of AZD1152, a specific inhibitor of Aurora B, at two different concentrations (0.1 and 10 µM). Nevertheless, no differences were detected in both parameters by blocking Aurora B activity, suggesting a specific role of Aurora A paralogue in this phenotype. Considering that drug treatment might be affecting the viability of CD8^+^ T lymphocytes, the toxicity of the inhibitors *per se* to CD8^+^ mouse T cells was tested at the incubation time (6 h) and concentrations previously used (10 µM for MLN8237, 1 µM for Aurora A Inhibitor I and 0.1 and 10 µM for AZD1152) without detecting any significant effects on cell viability (Supp. Fig. 2).

These results highlight the crucial role of Aurora A in CD8^+^ T cell “killing” capacity. Moreover, this role seems to be specific of Aurora A compared to Aurora B, despite their structural similarities.

### Aurora A controls the expression of cytotoxic genes and the TCR signal transduction cascade in CD8^+^ T cells

**Figure 2:**
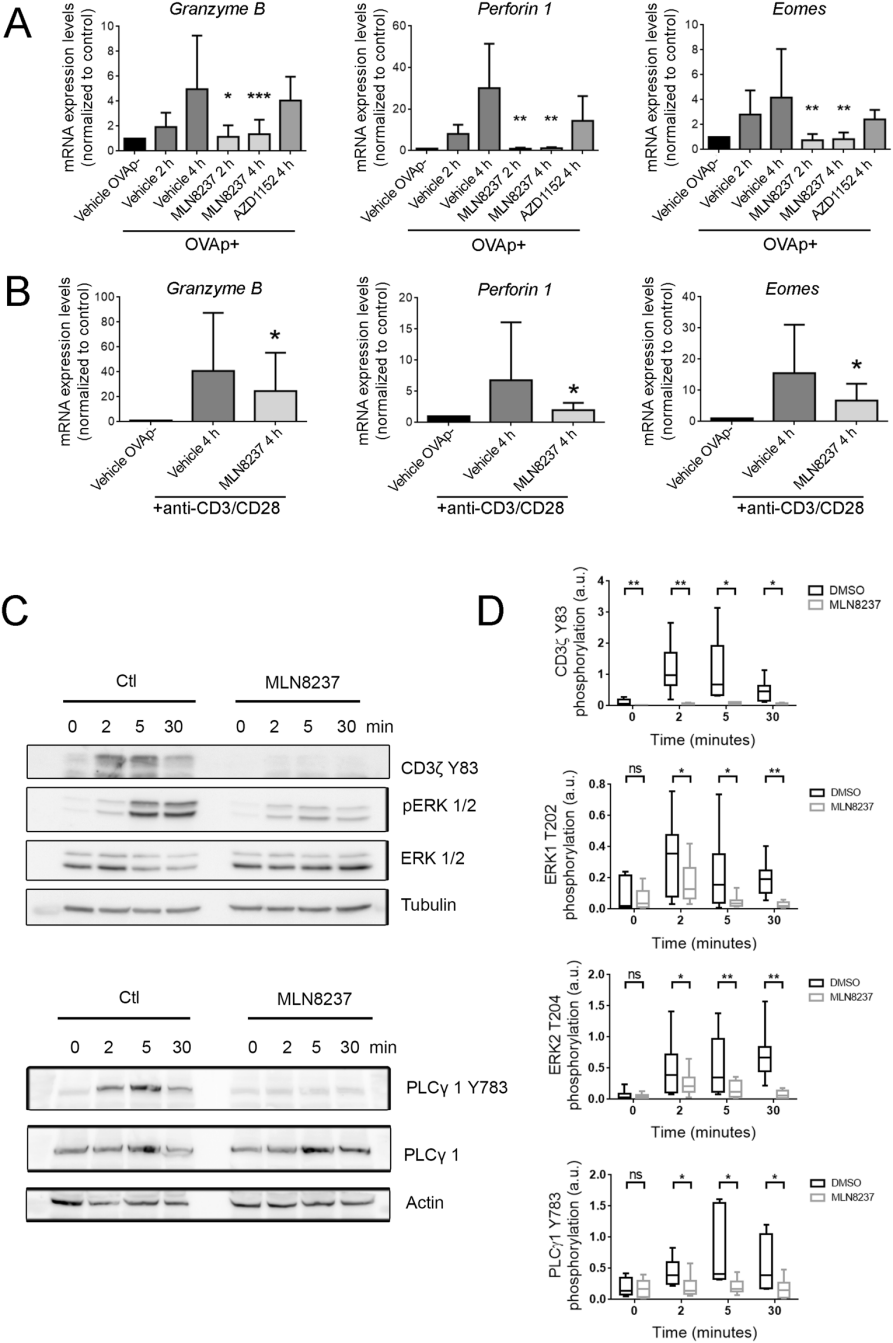
Aurora A enhances cytotoxic gene expression by driving TCR signalling. **(A**) mRNA levels of the indicated genes in mouse OTI-CD8^+^ T cells pretreated with vehicle, MLN8237 (10 µM) or AZD1152 (100 nM) and activated for 2 or 4 h with OVAp-charged or uncharged EL4 cells (10:1, T cell: target ratio; n = 9 mice samples, pair t-test or Wilcoxon signed rank test). **(B)** mRNA levels of the indicated genes in human CD8^+^ T cells pretreated with vehicle or MLN823 (10 µM) and activated for 4 h with anti-CD3/CD28 coated plates (n = 7 human samples, Wilcoxon signed rank test). **(C)** Immunoblots of the indicated molecules from extracts of mice OTI-CD8^+^ T cells pretreated with vehicle or MLN8237 (10 µM) and activated for the indicated times with OVAp-charged EL4 cells. Uncropped images of the gels are included in Supp. Fig. 3. (**D**) Quantification of the immunoblots is shown as in (**C**). (n = 10 and 6 samples, Wilcoxon signed rank test) P-value: < 0.05*, < 0.01**; < 0.001***. Mean ± s.d.

The induction of essential genes for effector CD8^+^ T cells cytotoxic activity (*eomes*, *perforin* and *granzyme B*) was next analysed. Assessment of mRNA expression after stimulation of MLN8237 or vehicle-treated mouse CD8^+^ T lymphoblasts with OVAp-pulsed EL4 cells for 2 and 4 h showed that Aurora A blockade led to a significant impairment in the induction of *eomes*, *perforin1* and *granzyme B* (Fig. 2A). As a control, cells were equally treated with Aurora B inhibitor (AZD1152), however, no significant changes were observed in the expression of these genes. Likewise, when human CD8^+^ T lymphoblasts gene expression pattern was analysed after stimulation of MLN8237 or vehicle-treated cells with anti-CD3/CD28 monoclonal antibodies, human *granzyme B*, *perforin1* or *eomes* gene upregulation was similarly decreased by Aurora A blockade (Fig. 2B).

To gain insights into the mechanism of Aurora A effect on signalling pathways of CTLs activation, the kinetics of phosphorylation of molecules implicated in the TCR activation cascade (CD3ζ, PLCγ1 and ERK1/2) was determined in extracts from vehicle (Ctl) or MLN8237-treated mouse CD8^+^ T lymphoblasts activated with OVAp-pulsed EL4 cells. Immunoblot analysis of phosphorylation showed a significant impairment in the activation of CD3ζ, PLCγ1 and ERK1/2 (Fig. 2C,D).

### Aurora A inhibition reduces CD8^+^ T cells cytotoxic *in vivo* response against *Vaccinia* infection

**Figure 3:**
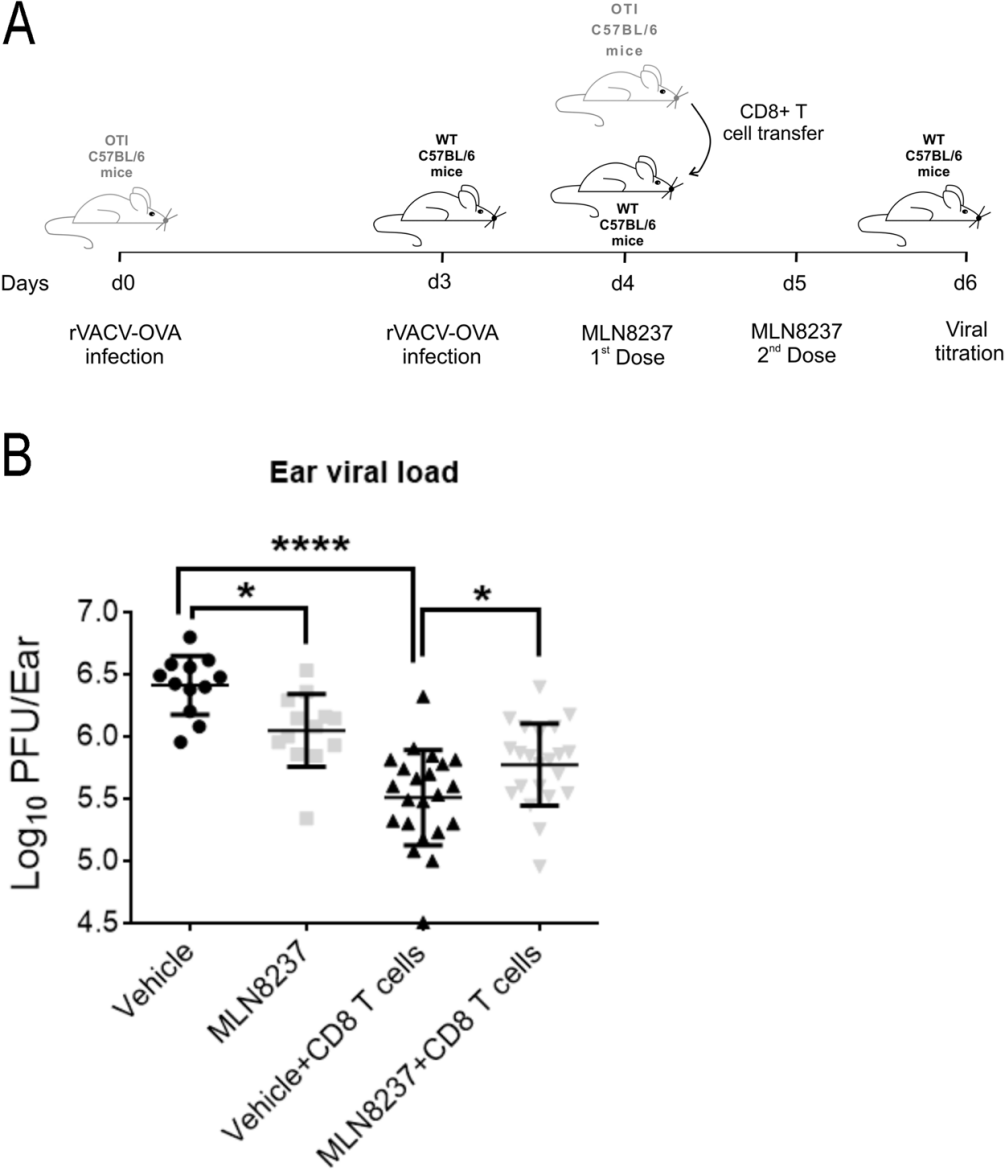
Aurora A boosts CD8^+^ T cell mediated antiviral response. (**A**) Schematic representation of the *in vivo* procedure. OTI mice were infected with rVACV-OVA and, after 4 days, their CD8^+^ T cells were transferred to preinfected WT C57BL/6 mice. WT mice were treated twice with the vehicle or MLN8237 and, after 2 days, euthanised for ear viral titration. (**B**) Ear viral load from vehicle (10% 2-hydroxypropyl-2-cyclodextrin/1% sodium bicarbonate) or MLN8237 (2 doses, 30 mg/kg by oral gavage) treated WT C57/BL6 mice, infected with rVACV-OVA for 3 days and adoptively transferred or not with OTI-CD8^+^ effector T cells. Ear viral load was quantified as the Log_10_ of plaque forming units (p.f.u) per ear. (n = 6 samples for mice without OTI transference and 11 samples for adoptive transference, both ears were quantified for each mouse; one way ANOVA with Turkey´s multiple comparison test). P-value: < 0.05*; < 0.0001****. Mean ± s.d.

In order to assess the influence of Aurora A inhibition in an *in vivo* infection model, we generated effector CD8^+^ T cells by infecting OTI mice with rVACV-OVA for 4 days. Next, we adoptively transferred these cells into MLN8237 or vehicle treated wild-type (WT) mice pre-infected with rVACV-OVA. We tested the cytotoxic activity of transferred OTI CTLs against vaccinia by measuring the viral load from both infected ears after 48 h (Fig. 3A). OTI CD8^+^ T cell transfer clearly reduced viral replication. Moreover, viral titration showed an increased number of viral plaques in those mice reconstituted with CTLs and treated with Aurora A inhibitor (*grey triangles*), in comparison with those with the CTLs and vehicle-treated (*black triangles*), indicating that Aurora A blockade leads to a defect in CD8^+^ T cells cytotoxic response against the virus (Fig. 3B). Conversely, MLN8237-treated infected mice that have not been adoptively transferred with OTI CD8^+^ T cells (*grey squares*), included as control, showed a decrease in viral titration compared to the vehicle-treated ones (*black squares*) (Fig. 3B). This suggests that Aurora A inhibition by MLN8237 might be interfering with viral replication *per se* or enhancing the innate immune clearance of the virus. Moreover, since CD8^+^ T cells transfer leads to a viral content still higher in the presence of MLN8237 than in vehicle treated mice, the reduction of the cytotoxic activity by Aurora A inhibition would be even stronger than the one detected.

These results show that Aurora A acts as a key molecule in the effector function of cytotoxic T lymphocytes, both in mice and human. We have previously described, in CD4+ T cells, the molecular mechanisms underlying Aurora A role on TCR pathway and microtubule dynamics^6^. However, in this work we have focused in the physiological effect of Aurora A blockade in CTL-activity during a global immune response. We have discovered a key role of Aurora A on CD8^+^ T cell *in vitro* cytotoxic capacity and cytotoxic gene expression and on the ability of CD8^+^ T cells to respond against infectious agents such as vaccinia virus. Taking into account the importance of Aurora A during cell division^4^ and the importance of the centrosome for lytic granule polarization and release in T cells^12,13^, Aurora A might be blocking the proper movement of lytic granules towards the IS, and therefore reducing their killing capacity.

Aurora A is important for both CD4^+^ T cell physiology^6^ and GVHD outcome^7,8^. This study supports its involvement on another relevant function of the adaptive immune response, namely CD8^+^ CTL-mediated cytotoxicity, which is crucial for the development of a proper global immune response.

T cell deficiency in cancer patients, mainly due to antineoplastic drugs, enhances patients susceptibility to infections^14,15^. Since some Aurora A inhibitors are being currently tested in the treatment of different types of malignancies^16^, Aurora A impairment of T cell cytotoxic response in infectious diseases would increase patients susceptibility to develop opportunistic infections. In fact, since it has been described that non-oncogenic acute infections promote tumor growth by sequestering and exhausting CTLs coming from the tumor^17^, it would be interesting to determine this possible side effect during antitumor treatments with Aurora A inhibitors.

Finally, considering the involvement of autoreactive CD8^+^ T cells in the generation of tissue damage on some autoimmune disorders, such as diabetes^18^, the results from this work unveil new applications for Aurora A inhibitors, such as the blockade of effector CTLs in order to prevent tissue damage and, thus, improve the outcome of these disorders.

## Materials and Methods

### Reagents and antibodies

The antibodies used in this study were anti-CD3ζ Y83 from Abcam; anti-α-Tubulin and anti-β-Actin from Sigma; anti-ERK1/2 T202/Y204 from Calbiochem; anti-ERK, anti-PLCγ1, anti-PLCγ1 Y783 from Cell Signaling Tech; anti mouse-CD8-APC and anti-CD69-FITC and anti-human IFNγ-FITC, anti-CD3ε (HIT3a) and anti-CD28 from BD Biosciences, anti-mouse and anti-human CD107a-APC and anti-human CD8-PE from Tombo Biosciences. Ovoalbumin peptide (SIINFEKL) was synthesized by LifeTein. The Aurora A inhibitors (MLN8237, 10 µM, and Aurora A Inhibitor I, 1 µM) and the Aurora B inhibitor (AZD1152, 10 µM and 100 nM) were from Selleckchem and they were dissolved in DMSO (vehicle). Horseradish peroxidase (HRP)-conjugated secondary antibodies for immunoblot (anti-rabbit, mouse or goat IgG + IgM) were from Pierce-Thermofisher Scientific. CFSE was from Invitrogen, Ghost Dyes (Red 780 and Violet 510) were from Tombo Biosciences. Fixation and permeabilization reagents were from eBiosciences. Murine IL-2 was from StemCell.

### Cells

The murine lymphoblastoid T cell line EL4 was cultured in RPMI 1640 + GlutaMAX-I + 25 mM HEPES (Gibco-Invitrogen) supplemented with 10% foetal bovine serum (Hyclone).

Human peripheral blood mononuclear cells (PBMCs) were isolated from buffy coats, obtained from healthy donors, by separation on a Biocoll gradient (Biochrom) according to standard procedures. To generate polyclonal cytotoxic T cells, CD8^+^ T cells were purified using MojoSort purification kit (Biolegend) and activated with anti-CD3/CD28 coated plates (5 and 3 μg/ml respectively). After two days, media was supplemented with IL-2 (50 U/ml) every 2 days for a time period of 8 days. These studies were performed according to the principles of the Declaration of Helsinki and approved by the local Ethics Committee for Basic Research at the Hospital La Princesa (Madrid); informed consent was obtained from all human volunteers.

### Mice

Male C57/BL6 wild-type mice and TCR (Vα2, Vβ5) transgenic mice (OTI), age 8–12 weeks, were housed in the pathogen-free animal facility of the Centro Nacional de Investigaciones Cardiovasculares Carlos III (Madrid) in accordance with the animal care standards of the institution. Animal experiments were approved by the local ethics committee and the Spanish Ministry of Agriculture and Fisheries, Food and Environment. All animal procedures conformed to EU Directive 86/609/EEC and Recommendation 2007/526/EC regarding the protection of animals used for experimental and other scientific purposes, enforced in Spanish law under Real Decreto 1201/2005.

T cells were obtained from single-cell suspensions of the spleen and lymph nodes from OTI mice. In order to generate CD8^+^ T lymphoblasts responsive to ovalbumin peptide (OVAp; SIINFEKL OVA_257–264_), cells were incubated 48 h with OVAp (10 ng/ml) and then, IL2 (50 U/ml) was added for a period of 5 additional days. CD8^+^ T lymphoblasts were cultured in RPMI 1640 + GlutaMAX-I + 25 mM HEPES (Gibco-Invitrogen) supplemented with 10% foetal bovine serum, 50 IU/ml penicillin, 50 µg/ml streptomycin (Gibco) and β-Mercaptoethanol (50 μM, Sigma).

### *In vitro* cytotoxicity assay

For cytotoxicity assay, EL4 cell line was used as target cells in a coculture with mice CD8^+^ T cells. Half of the EL4 cells were incubated with 1 µM CFSE (20 min) and pulsed with 1 µM OVAp (2 h) while the other half were incubated with 0.1 µM CFSE (20 min) alone. Both CFSE-stained populations were mixed (1:1) and cocultured with different ratios of mice CD8^+^ T cells during 6 h at 37 °C in technical triplicates. CD8^+^ T cells were previously treated with vehicle (DMSO), MLN8237, Aurora A Inhibitor I or AZD1152-pretreated for 30 min. After the 6 h of incubation, cells were stained with Red 780-Ghost Dye (Tombo Biosciences) in order to exclude death cells, and anti-CD8. Alive EL4 cells were monitored by flow cytometry, comparing the percentages of pulsed *vs* unpulsed cells. The percentage of specific lysis was calculated as follows:

$$100\times [1-( \% \,{\rm{of}}\,1{\rm{\mu }}{\rm{M}}\,{\rm{CFSE}}\,{\rm{cells}}/ \% \,{\rm{of}}\,0.1\,\mu {\rm{M}}\,{\rm{CFSE}}\,{\rm{cells}})].$$

### Degranulation assay

For mice, EL4 cells were pulsed with 1 μM of OVAp (2 h) and then cocultured with mouse OTI CD8^+^ T cells for 6 h at 37 °C. For human, CD8^+^ T cells were activated for 6 h with anti-CD3/CD28 coated plates (10 and 8 μg/ml, respectively) at 37 °C. Both cocultures were conducted in the presence of monensin (5 µM) and anti-CD107a – APC antibody in technical triplicates. Meanwhile, vehicle (DMSO), MLN8237 (10 µM) or AZD1152 (0.1 and 10 µM) were applied during the degranulation process. Finally, cells were stained with a viability staining (Violet 510 Ghost Dye) and anti-CD8. To quantify cell degranulation, the expression of the lysosomal marker CD107a was measured alone or in combination with IFNγ production (human samples). All flow cytometry data were analyzed with FlowJo software (TreeStar).

### Quantitative real-time PCR

RT-PCR was performed with 1 µg of total RNA isolated with Trizol RNA reagent (Invitrogen, Eugene, OR, USA). For mice samples, RNA was obtained from vehicle, MLN8237 (10 µM) or AZD1152-pretreated (0.1 µM) mice CD8^+^ T lymphoblasts cocultured for 2 or 4 h with OVAp-pulsed or unpulsed EL4 T cells (Ratio 10:1). For human samples, vehicle or MLN8237-pretreated CD8^+^ T cells were activated for 4 h with anti-CD3/CD28 (10 and 8 μg/ml, respectively) coated plates. mRNA levels of *Eomes*, *Granzyme B* or *Perforin1* were determined in technical triplicate using the Power SYBR Green PCR master mix (Applied Biosystems, Warrington, UK). Expression levels were normalised to the expression of a housekeeping gene (*Actin*). Primer sequences are listed in Supp. Table 1.

### CD8^+^ T cell activation, lysis and immunoblotting

For antigen stimulation, EL4 cells were pulsed with 1 μM of OVAp (2 h) and mixed with CD8^+^ T lymphoblasts for the indicated times. Where indicated, CD8^+^ T cells were pretreated with MLN8237 (10 μM) or vehicle (DMSO) for 30 min at 37 °C in medium prior to stimulation with EL4 T cells. Cells were centrifuged at low speed and let to conjugate for the indicated times at 37 °C. Cells were lysed in 50 mM Tris-HCl pH 7.5 containing 1% NP40, 0.2% Triton X-100, 150 mM NaCl, 2 mM EDTA, 1.5 mM MgCl_2_, and phosphatase and protease inhibitors. Lysates were spun at 20.000 × g (4 °C, 10 min) to remove debris and nuclei. Proteins were resolved by SDS–PAGE and transferred to nitrocellulose membranes. After blocking with TBS containing 0.2% TWEEN and 5% BSA, membranes were blotted with primary antibodies (o/n at 4 °C) and peroxidase-labelled secondary antibodies (30 min) and detected with the ImageQuant LAS-4000 chemiluminiscence & fluorescence imaging system (Fujifilm).

### Vaccinia virus infection and virus titration

Both ears from OTI mice were intradermally (i.d.) infected into the ear pinnae with 1.5 × 10^4^ PFU of rVACV expressing full-length OVA (a gift from J. W. Yewdell and J. R. Bennink. NIH, Bethesda, MD). Four days after infection, CD8^+^ T cells were isolated from the auricular lymph nodes and transferred to recipient WT mice, previously i.d. infected with OVA-VACV (36 h). In parallel, either Aurora A inhibitor MLN8237 (30 mg/kg) or vehicle (10% 2-hydroxypropyl-2-cyclodextrin/1% sodium bicarbonate) were administered, via oral gavage, to the mice. MLN8237 or vehicle administration was repeated after 24 h of CD8^+^ cell transference. For virus titration, the infected ears were mechanically disaggregated in 1 ml of PBS. After 2 freeze-thaw cycles and sonication, serial dilutions of ears homogenates were added to CV-1 cells monolayers seeded in 24 well plates. Cristal violet was used to stain them after 24 h and the number of plaques was multiplied by the reciprocal of sample dilution and converted to log_10_ of p.f.u/Ear^19^.

### Statistical analysis

First, a Shapiro-Wilk normality test was applied to determine the application of the corresponding parametric or non-parametric tests. Friedman or ANOVA t-test was used for grouped analysis while for dependent samples a paired analysis was used; either paired t-test (parametric) or Wilcoxon test (non-parametric). Analysis was performed with GraphPad Prism.

## Supplementary information

Supplementary Information
