# Supplementary material for: Aurora A controls CD8+ T cell cytotoxic activity and antiviral response
Source: Sci Rep. 2019 Feb 18;9:2211. doi: 10.1038/s41598-019-38647-y (PMC6379542; doi:10.1038/s41598-019-38647-y)
Supplement: Supplementary file 1 — Supplementary Information [file 41598_2019_38647_MOESM1_ESM.pdf]

Aurora A controls CD8$^+$ T cell cytotoxic activity and antiviral response

Eugenio Bustos-Morán[1#], Noelia Blas-Rus[1#], Ana Álcaraz-Serna[1], Salvador Iborra[3], José González-Martínez[4], Marcos Malumbres[4], Francisco Sánchez-Madrid[1,2,5*].

1. Servicio de Inmunología, Hospital Universitario de la Princesa, Universidad Autónoma de Madrid, Instituto Investigación Sanitaria Princesa (IIS-IP), Madrid, Spain

2. Centro Nacional Investigaciones Cardiovasculares (CNIC), Madrid, Spain

3. Department of Immunology, Ophthalmology and ENT. Complutense University School of Medicine and 12 de Octubre Health Research Institute (imas12), Madrid, Spain.

4. Cell Division and Cancer group, Centro Nacional de Investigaciones Oncológicas (CNIO), Madrid, Spain

5. CIBERCV. Madrid

*** Corresponding Author:** Francisco Sánchez Madrid, Hospital Universitario de la Princesa (Immunology Department), Diego de León 62, 28006, Madrid (Spain). 915202307. fsmadrid@salud.madrid.org.

# These authors contributed equally to the work

Supplementary information

Supplementary Table 1. List of primer sequences

Gene	Species	Forward primer	Reverse primer
Granzyme B	Mouse	TCACAAGGACCAGCTCTGTCCT	GTTGGGTTGTCACAGCATGG
Perforin1	Mouse	GAGAAGACCTATCAGGACCA	AGCCTGTGGTAAGCATG
Eomes	Mouse	CCCCTATGGCTCAAATTCC	CCAGAACCACTTCCACGAA
Actin	Mouse	CAGAAGGAGATTACTGCTCTGGCT	TACTCCTGCTTGCTGATCCACATC
Granzyme B	Human	GGAGGCCCTCTTGTGTGTAA	ATTACAGCGGGGGCTTAGTT
Perforin1	Human	ATTCACCCTGTCCAAACTGC	GTGGGCAAAGAAGACAGAGC
Eomes	Human	CCTGAGGAGCAAGAGGTACG	CACATTGTAGTGGGCAGTGG
Actin	Human	ATCATGTTTGAGACCTTCAA	AGATGGGCACAGTGTGGGT

Supplementary Figure 1

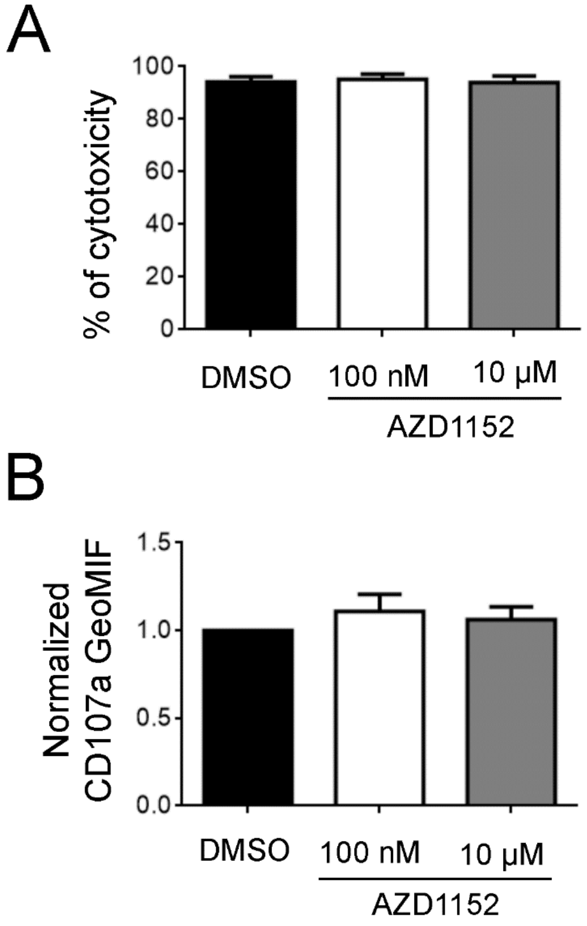

Supplementary Figure 1. Aurora B inhibition does not affect cytotoxicity and degranulation capacity. (A) Cytotoxic assay from, vehicle or AZD1152-treated, mouse OTI-CD8$^+$ T cells cocultured with unpulsed (CFSE-*low*) or OVAp-pulsed (CFSE-*high*) EL4 target cells for 6 h. Cocultures were performed at 5:1 (T cell *vs* target) ratio and the percentage of specific lysis was quantified as in the graph (n = 4, paired t-test, non significant) **(B)** Graph showing CD107a fluorescence intensity of, vehicle or AZD1152-treated, OTI-CD8$^+$ cells after 6 h of coculture with OVAp-pulsed EL4 in the presence of monensin. CD107a geometric mean was quantified and normalised to vehicle-treated as indicated in the graph (n=4 Wilcoxon signed rank test, non significant).

Supplementary Figure 2

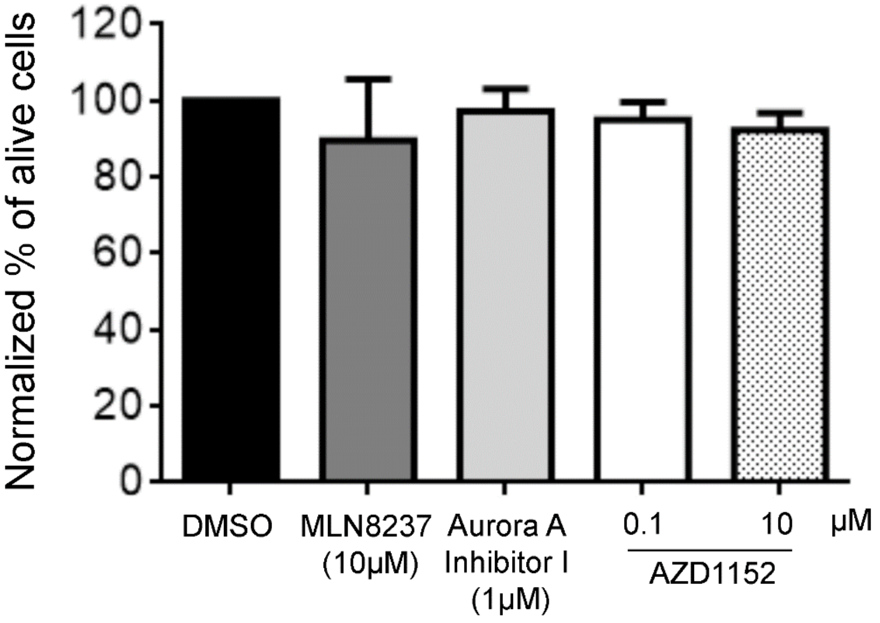

Supplementary Figure 2. Aurora A and Aurora B inhibitors do not reduce the viability of CD8+ mouse T cells. Graph showing the percentage of live cells in MLN8237 (10 µM), Aurora A Inhibitor I (1 µM) or AZD1152 (0.1 and 10 µM) treated mouse CD8+ T cells after 6 h. Percentages are normalised to those of vehicle (DMSO) treated cells. (n=4 Wilcoxon signed rank test, non significant).

Supplementary Figure 3

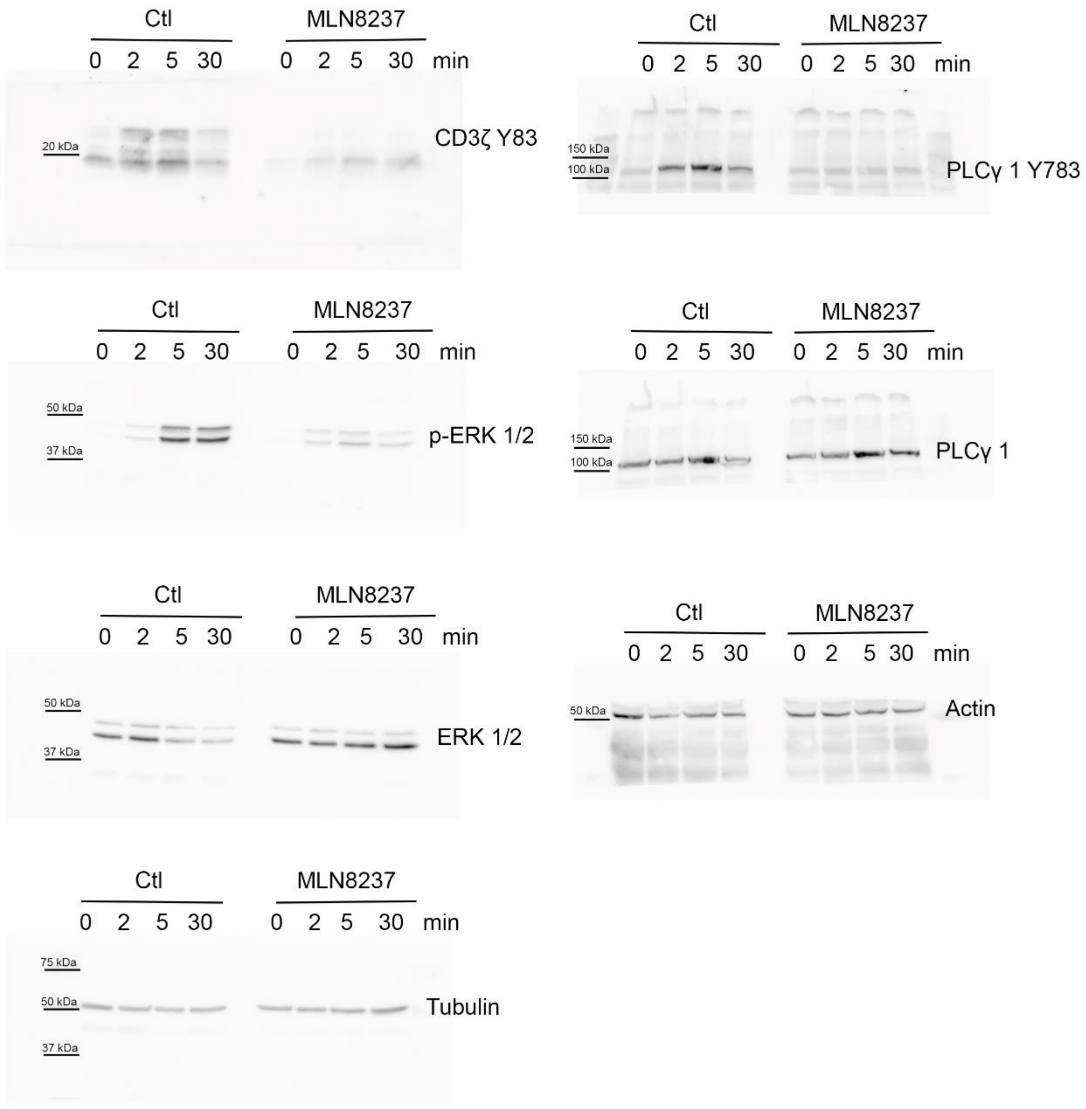

Supplementary Figure 3. Aurora A enhances cytotoxic lymphocytes TCR signalling. Uncropped images of the immunoblots of the indicated molecules from extracts of mice OTI-CD8^{+} T cells pretreated with vehicle or MLN8237 (10 µM) and activated for the indicated times with OVAp-charged EL4 cells.
